# Modified OMP Algorithm for Exponentially Decaying Signals

**DOI:** 10.3390/s150100234

**Published:** 2014-12-24

**Authors:** Krzysztof Kazimierczuk, Paweł Kasprzak

**Affiliations:** 1 Centre of New Technologies, University of Warsaw, Banacha 2C 02-097, Poland; 2 Department of Mathematical Methods in Physics, Faculty of Physics, University of Warsaw, Pasteura 5, Warsaw 02-093, Poland; E-Mail: pawel.kasprzak@fuw.edu.pl

**Keywords:** compressed sensing, orthogonal matching pursuit, exponentially decaying signal, nuclear magnetic resonance, non-uniform sampling, Lorentzian peaks matching pursuit

## Abstract

A group of signal reconstruction methods, referred to as compressed sensing (CS), has recently found a variety of applications in numerous branches of science and technology. However, the condition of the applicability of standard CS algorithms (e.g., orthogonal matching pursuit, OMP), i.e., the existence of the strictly sparse representation of a signal, is rarely met. Thus, dedicated algorithms for solving particular problems have to be developed. In this paper, we introduce a modification of OMP motivated by nuclear magnetic resonance (NMR) application of CS. The algorithm is based on the fact that the NMR spectrum consists of Lorentzian peaks and matches a single Lorentzian peak in each of its iterations. Thus, we propose the name Lorentzian peak matching pursuit (LPMP). We also consider certain modification of the algorithm by introducing the allowed positions of the Lorentzian peaks' centers. Our results show that the LPMP algorithm outperforms other CS algorithms when applied to exponentially decaying signals.

## 1. Introduction

Compressed sensing (CS) theory provides the methods to solve an underdetermined linear equation ***y*** = ***Ax*** within a set of sparse vectors *x* ∈ ℂ*^n^*. The unconstrained linear equation for an *m* × *n* matrix ***A*** of a maximum rank may have infinitely many solutions for *m* < *n*. However, the number of sparse solutions happens to be finite. Vectors in ℂ*^n^* having at most *l* ∈ ℕ non-zero coordinates are referred to as the *l*-sparse vectors.

All *l*-sparse vectors fulfilling the equation ***y*** = ***Ax*** may be found by considering $\left(\begin{array}{c} n\\ l \end{array}\right)$ linear sub-problems of solving ***y*** = ***Ax*** with the constraint ***x*** ∈ ℂ*_I_*, where ℂ*_I_* ⊂ ℂ^n^ denotes the subspace of vectors supported by I ⊂ [1,*n*]. This provides all l-sparse solutions, but the overall problem was proven to be NP-hard. Remarkably, the sparsest vector ***x̂*** solving ***y*** = ***Ax*** may be found as the solution of the *l*_1_-minimization problem:

$$\hat{x}=\underset{y=Ax}{\mathrm{argmin}}{\left\|x\right\|}_{1}$$

where ‖ ***x*** ‖ _1_ denotes the *l*-norm of ***x***. The *l*-norm convexity and the linear constraint ***y*** = ***Ax*** allow the application of linear programming algorithms, as long as ***A*** has a restricted isometry property; see [1].

A number of CS algorithms has been proposed, including: iterative soft thresholding (IST) [2], orthogonal matching pursuit (OMP) [3], compressive sampling matching (COSAMP) [4], stage-wise orthogonal matching pursuit (StOMP) [5] and iterative re-weighted least squares (IRLS) [6]. Inspired by the greedy CS algorithm, OMP, and motivated by a case of nuclear magnetic resonance (NMR) spectroscopy, we propose an approach that is designed specifically to recover a signal being a combination of exponentially decaying components. The spectrum of such a signal consists of Lorentzian peaks. The algorithm matches a single peak in each of its iterations, and thus, the name Lorentzian peak matching pursuit (LPMP) is proposed. The peaks' centers and heights are found as in the OMP algorithm, whereas the widths of the peaks are determined in a novel way.

## 2. NMR Spectroscopy and CS

The sources of the free induction decay (FID) signal measured in NMR spectroscopy are magnetic moments of atomic nuclei with non-zero spin, e.g., ^1^H,^13^C,^15^N,^19^F, that undergo coherent precession when excited by radio-frequency irradiation in the presence of a high static magnetic field [7]. The time dependence of an ideal FID signal *f*(*t*) is given by the following linear combination:

(1) $$f\left(t\right)=\sum_{k=1}^{N}a_{k}f_{k}\left(t\right)$$

of the exponentially decaying oscillations:

$$f_{k}\left(t\right)=\{\begin{array}{cc} e^{-2\pi \gamma_{k}t}e^{2\pi \iota \omega_{k}t} & t\geq 0\\ 0 & t<0 \end{array}$$

where *N* is a number of nuclei groups differing in resonance frequency; *a_k_* are amplitudes (the (x0221D) number of nuclei in the *k*-th group); *γ_k_* is a relaxation rate for the *k*-th group; and ω*_k_* is an oscillation frequency of the *k*-th group.

The precession frequencies (and thus, the frequencies of the emitted FID signal) are in the order of MHz, but their band is very narrow, typically a few kHz. In such a narrow range, tens or even hundreds of peaks are present. Often, the spectral resolution (peak width compared to the difference in resonance frequencies) is too low to resolve peaks and, thus, makes an analysis difficult. Fortunately, the peak dispersion can be improved by the acquisition of the multidimensional NMR signal [8] where additional (“indirect”) time dimensions are introduced. Peaks in the multidimensional NMR spectrum correspond to groups of nuclei coupled by some physical interaction. Using the above notation, the formula of the two-dimensional FID is:

(2) $$f\left(t_{1},t_{2}\right)=\sum_{k_{1},k_{2}=1}^{N}a_{k_{1},k_{2}}f_{k_{1}}\left(t_{1}\right)f_{k_{2}}\left(t_{2}\right)$$

where *t*_1_ is indirectly sampled time and *t*_2_ directly sampled time.

The reason for the application of CS in multidimensional NMR spectroscopy is a time-consuming sampling of the indirect dimensions of the FID signal (*t*_1_ in Equation (2)). There are three reasons for the lengthy acquisition. Firstly, each measurement point in the indirect dimension takes a few seconds of “real” time. Secondly, the sampling density has to fulfil the Nyquist theorem [9]. Finally, the sampling has to reach far into the indirect time domain to provide sufficient spectral resolution [10]. The latter, together with the Nyquist condition for the sampling density, means that, often, many thousands of sampling points have to be acquired. This makes an experiment unacceptably long (days in the case of three or four dimensions) or completely impossible in the case of higher dimensionality (5D and more). Thus, there is a need for alternative sampling approaches that allow one to save expensive spectrometer time preserving the spectral information. Most of them use the non-uniform sampling, where the major part of the points is not measured, but reconstructed after the experiment based on certain assumptions [11,12]. The CS, where the assumption is spectral sparsity, has been successfully applied in NMR exploiting the algorithms based on convex *l*_1_-norm or non-convex *l_p_*-norm minimization [13,14]. The history of the application of the “greedy” algorithm, OMP, in NMR is long; in fact, the algorithm was transferred from radio astronomy, where it existed under the name, CLEAN [15]. Direct application of CLEAN in NMR is possible [16], but far from being perfect, because of the aforementioned approximate sparsity of the signal. Thus, various modifications of CLEAN have been proposed [17-19]. Importantly, none of these works refer to OMP formalism and do not explain the modifications by the sparsity criteria. We propose an approach that directly exploits the fact that the NMR spectrum consists of a low number of Lorentzian peaks. Thus, LPMP is based on the sparsity concept, which is more relevant in the case of NMR than classical CS methods.

## 3. OMP Algorithm

Let us fix the notation. For *m,n* ∈ ℤ and *m* < *n*, we write [m, n] = {m, m + 1,…, n}. Let *m, n* ∈ ℕ. For matrix ***A*** ∈ M*_m×n_*(ℂ), its range and kernel are denoted ran ***A*** and ker ***A***, respectively. The Hermitian conjugate of ***A*** is denoted by A*. The number of non-zero coordinates of ***x*** ∈ *C^n^* is denoted by ‖***x***‖_0_, and supp ***x*** (the support of ***x***) denotes the set of ***x*** non-zero coordinates. Note that ‖***x***‖_0_ = # supp ***x*** (the cardinality of a set *I* is denoted by # *I*). We define *χ_n,l_* ⊂ ℂ*^n^*:

$$\chi_{n,l}=\left\{x\in {\text{C}}^{n}:{\left\|x\right\|}_{0}\leq l\right\}$$

OMP algorithm arose in the context of the sparse approximation problem; see [3]. In order to formulate the problem, let us fix a matrix ***A*** ⊂ M*_m×n_*(ℂ) satisfying ran ***A*** = ℂ*^m^*. The matrix ***A*** is in this context referred to as the dictionary matrix.

### Definition 1

Adopting the above notation, we say that ***x*** ∈ *χ_n,l_* is an optimal *l*-sparse approximate representation of a vector ***y*** ∈ ℂ*^m^* if:

$${\left\|y-Ax\right\|}_{2}=\min\left\{{\left\|y-Ax^{\prime }\right\|}_{2}:x^{\prime }\in \chi_{n,l}\right\}$$

We say that *y* is *l*-representable, if there exists a vector ***x*** ∈ *χ_n,l_*, such that ***y*** = ***Ax***.

For a subset *I* ⊂ [1, *n*] of cardinality *l*, we define a vector subspace ℂ*_I_* ⊂ ℂ*^n^*:

$${\text{C}}_{I}=\left\{x\in {\text{C}}^{n}:\text{supp}\left(x\right)\subset I\right\}$$

The restriction of ***A*** to ℂ*_I_* is denoted by ***A****_I_* : ℂ*_I_* → ℂ*^m^*.

### Definition 2

We say that ***A*** ⊂ M_*m*×*n*_(ℂ) is *l*-injective if ker ***A****_I_* = {0} for all *I* ⊂ [1, *n*], # *I* = *l*.

For an *l*-injective matrix ***A*** and *I* ⊂ [1, *n*], # *I* = l, we define:

(3) $$x_{1}=\underset{x^{\prime }\in {\text{C}}_{I}}{\mathrm{argmin}}{\left\|y-A_{I}x^{\prime }\right\|}_{2}$$

Since **x***_I_* is a vector satisfying **A***_I_x_I_* = ***y****_I_*, where ***y****_I_* is an orthogonal projection of ***y*** onto ran ***A****_I_*, we may see the uniqueness of ***x****_I_* for *l*-injective ***A***. Let us note that ***x*** ∈ *χ_n,l_* is an optimal *l*-sparse approximate representation of ***y*** ∈ ℂ*^m^* if:

$${\left\|y-Ax\right\|}_{2}\underset{\left\{I:\#I=l\right\}}{\min}{\left\|y-Ax_{I}\right\|}_{2}$$

Although l-injectivity of the matrix ***A*** does not exclude a vector ***x***′ ∈ *χ_n,l_*, ***x***′ ≠ ***x***, such that ‖***y*** — ***Ax***‖_2_ = ‖***y*** — ***Ax*′**‖_2_, we shall usually choose an optimal *l*-sparse approximate and denote it by ***x****_l_*.

The OMP algorithm provides a certain approximation of *x_l_* ∈ ℂ^n^, which we denote by $x_{l}^{\text{OMP}}$ Remarkably, $x_{l}^{\text{OMP}}$ is found on the basis of $x_{l-1}^{\text{OMP}}$ Denoting the $x_{l}^{\text{OMP}}$ support by $I_{l}^{\text{OMP}}$, the index i*_l_* ∈ [1, *n*] is given by:

$$i_{l}=\underset{i\in \left[1,n\right]}{\mathrm{argmax}}{\left|A*\left(y-Ax_{l-1}\right)\right|}_{i}$$

The OMP vector $x_{l}^{\text{OMP}}$ is defined as $x_{l}^{\text{OMP}}=x_{I_{l}^{\text{OMP}}}$; see Equation (3) for the definition of $x_{I_{l}^{\text{OMP}}}$. For the coherence, restricted isometry property (RIP) conditions that imply the equality $x_{l}^{\text{OMP}}=x_{l}$, we refer to [3] and [20], respectively. For the recovery limits of the OMP, see [21]. The OMP algorithm (Algorithm 1) is schematically described below.



**Algorithm 1** Orthogonal matching pursuit.

*Input*:
measurement matrix **A** ∈ M*_m×n_*(ℂ)measurement vector ***y*** ∈ ℂ*^m^*accuracy parameter *ε* > 0*Output:*
*x* ∈ ℂ*^n^**Initialization:*
*I* = ∅, ***x*** = 0The main loop:**while** ‖***y*** — ***Ax***‖_2_ ≥ *ε* **do**

$I=I\cup \left\{\underset{j\in \left[1,n\right]}{\mathrm{argmax}}\left|A*{\left(y-Ax\right)}_{j}\right|\right\}$
$x=\underset{\text{supp}\left(z\right)\subset I}{\mathrm{argmin}}{\left\|y-Az\right\|}_{2}$**end while**


## 4. Free Induction Decay Signals in NMR

The free induction decay signal in NMR consists of the combination of exponentially decaying oscillations (see Equations (1) and (2)). The signal can be multidimensional, but for the sake of simplicity, we will limit ourselves to the one-dimensional indirect part of the two-dimensional signal, corresponding to *f*(*t*) from Equation (1). Given the damping factor *γ* ≥ 0 and the oscillation frequency ω ∈ (x0211D), a single decaying oscillation is given by:

$$f\left(t\right)=\{\begin{array}{cc} e^{-2\pi \gamma t}e^{2\pi_{l}\omega t} & t\geq 0\\ 0 & t<0 \end{array}$$

Its Fourier transform:

$$\hat{f}\left(v\right)=\int_{\text{R}}f\left(t\right)e^{-2\pi lvt}dt$$

has the form of complex Lorentzian peak:

$$\hat{f}\left(v\right)=\frac{1}{2\pi }\frac{1}{\gamma -\iota \left(\omega -v\right)}$$

The FID signal is sampled at the rate given by the Nyquist theorem for the range cut off *ν* ∈ [–Ω, Ω]. In order to perform the NMR signal processing, the frequency resolution ΔΩ is introduced, where $\Delta \Omega =\frac{\Omega }{2N+1}$ and *N* ∈ ℕ. For any *l* ∈ [–*N,N*], we get the basic complex oscillation *e^2πιωlt^*, where $\omega_{l}=\frac{l\Omega }{2N+1}$. The NMR signals can be approximated by the finite combination of the basic complex oscillations:

$$f\left(t\right)\approx \sum_{l=-N}^{N}a_{l}e^{2\pi l\omega lt}$$

The approximation being $\frac{2N+1}{\Omega }$ -periodic works only for $0\leq t\leq \frac{2N+1}{\Omega }$. Assuming that the series $\sum_{l=-N}^{N}a_{l}e^{2\pi l\omega lt}$evaluated at $t_{k}=\frac{k}{\Omega }$ coincides with sampling points of *f, i.e., f*(*t_k_*) for *k* ∈ [0,2*N*], we get:

$$a_{l}=\frac{1}{2N+1}\sum_{k=0}^{2N}f\left(t_{k}\right)e^{-2\pi l\omega_{l}t_{k}}$$

Introducing the time unit $\frac{1}{\Omega }$ and the frequency unit $\frac{\Omega }{2N+1}$, we may enumerate the time and frequency by the integers [0, 2*N*] and [–*N, N*], respectively. Then, the relation between the peak intensities ***a*** and the time samples ***f*** is given by ***a*** = ***Ff***, where ***F*** is the Fourier transform matrix:

$$F_{kl}=e^{-2\pi lkl/2N+1}$$

*l* ∈ [–*N, N*] and *k* ∈ [0, *2N*]. In order to model the Lorentzian peaks within our framework, let us introduce the width peak unit Γ and a damping matrix ***C*** ∈ M_(2_*_N_*_+1)×(2_*_N_*_+1)_(ℂ):

$$C_{kk^{\prime }}=\delta_{kk^{\prime }}e^{-2\pi k\Gamma /\Omega }$$

for *k, k*′ ∈ [0, 2*N*]. Then, the Lorentzian peak of width Γ centered at *l* is represented by ***L***_l_ :

$$L_{l}=F^{-1}CF_{l}$$

where ***F****_l_* is the *l*-th column of ***F***. Similarly, the Lorentzian peak of the width *j*Γ centered at *l* is represented by:

(4) $$L_{l}^{j}=F^{-1}C^{j}F_{l}$$

Using the above notation, the FID signal is given by:

(5) $$f=\sum_{jl}b_{jl}FL_{l}^{j}$$

where ***b****_jl_* ∈ ℂ.

## 5. Lorentzian Peak Matching Pursuit

The spectrum ***f̂*** corresponding to FID signal (Equation (5)) is given by:

$$\hat{f}=\sum_{jl}b_{jl}L_{l}^{j}$$

We shall assume that *j* ∈ [0, *J*], which gives the maximal width *J*Γ and the width resolution Γ. Let *K* = {*k*_1_, *k*_2_,…, *k_m_*} ⊂ [0, 2*N*] be the sampling scheme, ***y*** ∈ ℂ*^m^* the measured signal ***y****_i_* = ***f***(*t_ki_*) and ***A*** = **F***_k_* the matrix consisting of rows of the Fourier matrix ***F*** enumerated by the elements of *K*.

The Lorentzian peak matching pursuit algorithm matches a Lorentzian peak $L_{l}^{j}$ in each of its iterations. In order to match a peak $L_{l}^{j}$ LPMP establishes first the peak's center *l*. The center *l*_1_ ∈ [–*N*, *N*] of the first peak is determined by:

$$l_{1}≔\underset{l\in \left[-N,N\right]}{\mathrm{argmax}}\left|{\left(A*y\right)}_{l}\right|$$

In order to determine the width of the first peak *j*_1_ ∈ [0, *J*], we find ***b****l*_1_,*_j_* ∈ ℂ, such that:

$${\left\|y-b_{l_{1},j}AL_{l_{1}}^{j}\right\|}_{2}=\min\left\{{\left\|y-zAL_{l_{1}}^{j}\right\|}_{2}:z\in \text{C}\right\}$$

Then, *j*_1_ ∈ [0, *J*] is a number satisfying:

$${\left\|y-b_{l_{1},j_{1}}AL_{l_{1}}^{j_{1}}\right\|}_{2}=\min\left\{{\left\|y-b_{l_{1},j}AL_{l_{1}}^{j}\right\|}_{2}:j\in \left[0,J\right]\right\}$$

and we put ${\hat{f}}_{1}=b_{l_{1},j_{1}}L_{l_{1}}^{j_{1}}$.

In the second step of the algorithm, we determine the second peak's center *l*_2_ by:

$$l_{2}=\underset{l\in \left[-N,N\right]}{\mathrm{argmax}}\left\{\left|A*{\left(y-A{\hat{f}}_{1}\right)}_{l}\right|\right\}$$

In order to determine the second peak's width *j*_2_ ∈ [0, *J*], we find ***b****_l_*_2_*_,j_*, ***b****_l_*_1_,*_j_*_1_ ∈ ℂ minimizing:

$${\left\|y-z_{2}AL_{l_{2}}^{j}-z_{1}AL_{l_{1}}^{j_{1}}\right\|}_{2}$$

where *z*_1_,*z*_2_ ∈ ℂ Defining *j*_2_ *E* [0, *J*] as a number satisfying:

$${\left\|y-b_{l_{2},j_{2}}AL_{l_{2}}^{j_{2}}-b_{l_{1},j_{1}}AL_{l_{1}}^{j_{1}}\right\|}_{2}=\min\left\{{\left\|y-b_{l_{2}}AL_{l_{2}}^{j}-b_{l_{1},j_{1}}AL_{l_{1}}^{j_{1}}\right\|}_{2}:j\in \left[0,J\right]\right\}$$

we put ${\hat{f}}_{2}=b_{l_{2},j_{2}}L_{l_{2}}^{j_{2}}+b_{l_{1},j_{1}}L_{l_{1}}^{j_{1}}$.

We begin the third step of the algorithm by determining the third peak's center *l*_3_:

$$l_{3}=\underset{l\in \left[-N,N\right]}{\mathrm{argmax}}\left|A*{\left(y-A{\hat{f}}_{2}\right)}_{l}\right|$$

Then, *j*_3_ and *l*_3_ are found as in the previous steps.

Note that in the *k*-th step of the algorithm, the peak's width *j_k_*Γ is found and not changed afterwards. The *b_lk_*,*_jk_* coordinate may, in turn, vary in the *n*-th step of the algorithm for *n* > *k*. The algorithm is performed until the accuracy threshold ‖***y*** – ***Ax***‖ < *ε* is achieved; for a different stopping criterion, see Section 5.1.

### 5.1. Stopping Criterion

If a number of Lorentzian peaks in the spectrum cannot be assumed *a priori*, then the stopping criterion for the LPMP becomes a crucial issue.

Notably, the dimension of a measurement vector *y* provides a mathematical bound for the number of iterations in LPMP. The implementation of the LPMP algorithm uses an inversion of a matrix, which ceases to be invertible when the number of peaks exceeds the number of measurements. In what follows, we shall introduce the stopping criteria that further restrict the number of iterations. One commonly used criterion is the threshold *ε* of accuracy used in Algorithm 2.

Let us denote the result of the *k*-th LPMP iteration by *f̂_k_*. The *k* + 1 iteration is performed if:

$${\left\|y-A{\hat{f}}_{k}\right\|}_{2}\leq \alpha {\left\|y-A{\hat{f}}_{k-1}\right\|}_{2}$$

where *α* ∈ [0,1] is an user-defined number. The criterion reflects the idea that the LPMP is continued as long as its actual iteration increases the explanation of the measured data by the factor *α* with respect to the previous iteration. In our simulations described in the next section, we put *α* = 0.98.

Alternatively, the noise amplitude *σ* can be used for a stopping criterion. In this case, LPMP stops on the *k*-th iteration if |***f****^_k_(l_k_)*| ≤ *σ*.



**Algorithm 2** Lorentzian peak matching pursuit.

*Input:*
measurement matrix ***A*** ∈ M*_K_*_×2_*_N_*_+1_(ℂ)measurement vector ***y*** ∈ ℂ*^K^*accuracy *ε* > 0Lorentzian peaks $L_{l}^{i}\in {\text{C}}^{2N+1}$ where *j* ∈ [0, *J*], *l* ∈ [−*N, N*]*Output:*
***f****^***_LPMP_**∈ ℂ^2^*^N^*^+1^*Initialization:*
***f****^***_LPMP_**= 0, ***j*** = 0, ***b*** = 0, *i* = 0The main loop:**while** ‖***y*** − ***Af****^***_LPMP_**‖_2_ ≥ *ε* **do**
*i* = *i* + 1
$l_{i}≔\underset{l\in \left[-N,N\right]}{\arg\max}\left|A*{\left(y-A{\hat{f}}_{\text{LPMP}}\right)}_{l}\right|$
$\left(b_{l_{i},j,}b_{l_{i-1},j_{j-1},\cdots ,}b_{l_{i},j_{1}}\right)=\underset{z\in {\text{C}}^{i}}{\mathrm{argmin}}{\left\|y-A\left(z_{i}L_{l_{i}}^{j}+z_{i-1}L_{l_{i-1}}^{j_{i-1}}+\ldots z_{1}L_{l_{1}}^{j_{1}}\right)\right\|}_{2}$
$j_{i}=\underset{j\in \left[0,J\right]}{\mathrm{argmin}}{\left\|y-A\left(b_{l_{i},j}L_{l_{i}}^{j}+b_{l_{i-1},j_{i-1}}L_{l_{i}-1}^{j_{i-1}}+b_{l_{1},j_{1}}L_{l_{1}}^{j_{1}}\right)\right\|}_{2}$
${\hat{f}}_{\text{LPMP}}=b_{l_{i},j_{i}}L_{l_{i}}^{j_{i}}+b_{l_{i-1},j_{i-1}}L_{l_{i-1}}^{j_{i-1}}+b_{l_{1},j_{1}}L_{l_{1}}^{j_{1}}$**end while**


### 5.2. Mask

NMR experiments are often performed in series, with some or all peaks preserving their positions in at least some of the dimensions. This fact has been exploited to restrict the allowed peak frequencies and to improve the results of NUS (non-uniform sampling) reconstruction, e.g., in hyperdimensional spectroscopy [22], the SIFT method [23] or high-dimensional spectra [24]. It is easy to use this information in the LPMP algorithm by introducing the subset Mask ⊂ [−*N, N*] of admissible peak centers in the frequency domain. In what follows, we give a detailed description of the masked LPMP algorithm (Algorithm 3), taking into an account the stopping criterion described in Section 5.1.


**Algorithm 3** Lorentzian peak matching pursuit with a mask.

*Input:*
measurement matrix ***A*** ∈ M*_K_*_×2_*_N_*_+1_(ℂ)measurement vector ***y*** ∈ ℂ*^K^*stopping parameter *α* > 0Lorentzian peaks $L_{l}^{i}\in {\text{C}}^{2N+1}$ where *j* ∈ [0, *J*], *l* ∈ [−*N*, *N*]Mask ⊂ [−*N, N*]*Output:*
***f****^* **_LPMP_** ∈ ℂ^2^*^N^*^+1^*Initialization:*
***f****^* **_LPMP_** = 0, ***j*** = 0,***b*** = 0,*i* = 0,*δ* = ‖***y***‖_2_The main loop:**while** ‖***y*** – ***Af****^***_LPMP_**‖_2_ ≤ α*δ* or *i* = 0 **do**
*δ* = ‖***y*** – ***Af****^*‖_2_*i* = *i* + 1
$l_{i}≔\underset{l\in \text{Mask}}{\arg\max}\left|A*{\left(y-A{\hat{f}}_{\text{LPMP}}\right)}_{l}\right|$
$\left(b_{l_{i},j,}b_{l_{i-1},j_{i-1}},\ldots ,b_{l_{1},j_{1}}\right)=\underset{z\in {\text{C}}^{i}}{\mathrm{argmin}}{\left\|y-A\left(z_{i}L_{l_{i}}^{j}+z_{i-1}L_{l_{i-1}}^{j_{i-1}}+\ldots +z_{1}L_{l_{1}}^{j_{1}}\right)\right\|}_{2}$
$j_{i}=\underset{j\in \left[0,J\right]}{\mathrm{argmin}}{\left\|y-A\left(b_{l_{i},j}L_{l_{i}}^{j}+b_{l_{i-1},j_{i-1}}L_{l_{i-1}}^{j_{i-1}}+\ldots b_{l_{1},j_{1}}L_{l_{1}}^{j_{1}}\right)\right\|}_{2}$
${\hat{f}}_{\text{LPMP}}=b_{l_{i},j_{i}}L_{l_{i}}^{j_{i}}+b_{l_{i-1},j_{i-1}}L_{l_{i-1}}^{j_{i-1}}+\ldots +b_{l_{1},j_{1}}L_{l_{1}}^{j_{1}}$**end while**


## 6. Results and Discussion

In this section, we present the results of the LPMP performance tests. Extensive simulations on different levels of sampling sparsity provided a quantitative characterization of the method. As an example of an application, we have chosen the challenging NOESY (nuclear Overhauser effect spectroscopy) experiment [25] with a high dynamic range of peak intensities.

The parameters of the synthetic spectrum used in the simulations are given in Table 1. The spectral parameters were chosen to simulate various experimental scenarios, *i.e.*, (I) peak widths differ from peak to peak, which corresponds to the differences in relaxation rates in the case of the NMR experiment; (II) the range of intensities is high; (III) two of the peaks overlap; and (IV) the peaks' centers and widths are off-grid.

**Figure 1.**
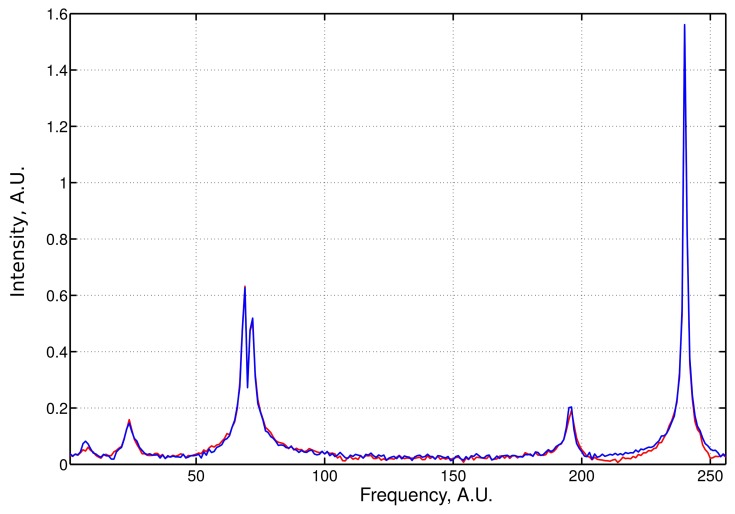
Example of the Lorentzian peak matching pursuit (LPMP) recovery. The size of the spectrum is 256. The number of randomly chosen samples is 120. The result of LPMP recovery (blue line) is compared with the original spectrum (red line).

**Figure 2.**
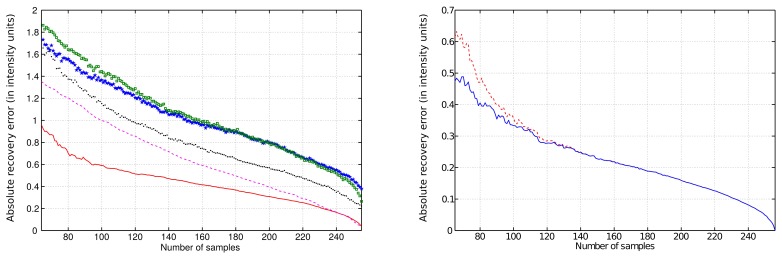
Quality of the reconstructions of the spectrum from Figure 1. (**Left**) The comparison of the performance of various algorithms: LPMP (solid line), iterative soft thresholding (IST) (dashed line), stage-wise orthogonal matching pursuit (StOMP) (dotted line), compressive sampling matching (COSAMP) (stared line) and orthogonal matching pursuit (OMP) (squared line). (**Right**) The performance of the masked LPMP algorithm (solid line) compared with the performance of non-masked LPMP algorithm (dashed line). The absolute recovery errors (in intensity units) are plotted vs. the number of sampling points.

An example of the **LPMP** recovery of an NMR spectrum based on 120 sampling points drawn at random from a 256-point full grid is presented in Figure 1. The signal was contaminated with 5% of noise. We performed systemic simulations with a varying sampling level. For each level, 50 different sampling schedules were generated. Denoting the frequency spectrum vector obtained from the full sampling by ***f̂*** and the **LPMP** recovery by ***f̂*_LPMP_**, we compute the recovery error ‖***f̂***–***f̂*_LPMP_**‖, and then, we average it over 50 sampling distributions. The LPMP performance is depicted in Figure 2 (left), where it is also compared with other popular CS algorithms: IST, OMP, COSAMP and StOMP. For all sampling levels, LPMP is revealed to be superior to other CS algorithms.

In order to test the masked version of LPMP algorithm, we introduced the mask of ±5 frequency points around the peaks' centers. The simulation results are presented in Figure 2 (right). As expected, masking has a more pronounced effect on the low sampling levels.

**Figure 3.**
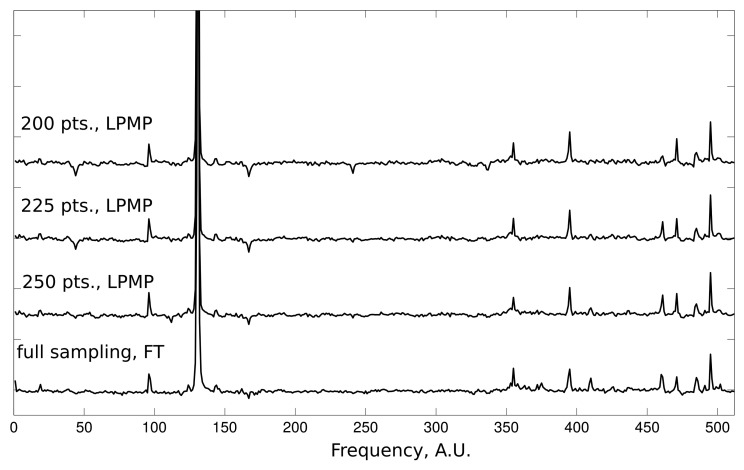
1D indirect dimension slice from 2D NOESY spectrum: LPMP-reconstruction from 200, 225 and 250 samples out of a 512-point full grid compared with a fully sampled spectrum.

The qualitative aspects of LPMP reconstruction can be evaluated on the data from the 2D NOESY experiment. Each reconstructed 1D slice of the 2D NOESY spectrum contains one dominating peak accompanied by a group of smaller peaks. Such a case of large intensity range is very challenging for CS methods. In Figure 3, we present the results of the LPMP spectrum recovery obtained on the basis of 200, 225 and 250 random measurements of the corresponding FID signal of length 512. The 2D NOESY spectrum was recorded on a 600-MHz Varian UNITY Inova spectrometer at 25 °C using a sample of human ubiquitin protein (1 mM concentration).

In order to explain the effectiveness of LPMP algorithm in the NMR context, let us emphasize the key difference between the OMP and LPMP methods. OMP tries to obtain the frequency-domain representation having the minimal number of non-zeros, which cannot be exact, even for a single Lorentzian peak. LPMP improves OMP by replacing every determined spectral non-zero with the Lorentzian peak of the appropriate width. Roughly speaking, this corresponds to the concept of sparsity in the sense of a number of Lorentzian peaks.

## 7. Conclusions

Our results show that LPMP is superior to the generic CS methods when applied to exponentially decaying signals. Comparison with OMP, StOMP, IST and COSAMP revealed that the assumption of the Lorentzian spectral line shape improves the quality of the reconstruction with LPMP. Moreover, its performance can be further improved by limiting (“masking”) the support of a signal. We believe that this method will find applications in NMR spectroscopy. Other fields of possible application may include different kinds of spectroscopy, wireless communications, sonar and radar. In fact, reconstruction of any non-uniformly sampled exponentially-damped signal can benefit from the application of LPMP.

## Figures and Tables

**Table 1.** Peak coordinates for the simulation presented in Figure 1.

**Center**	**5.6**	**22.7**	**67.8**	**70.4**	**194.5**	**239.25**
width	5.2	5.4	2.6	2.8	4	1.5
